# Laryngeal mucinous adenocarcinoma in a 45-year-old woman: a case report

**DOI:** 10.1097/MS9.0000000000002942

**Published:** 2025-02-13

**Authors:** Gabrielle Angela G. Mercado, Tuan-Jen Fang, Li-Yu Lee, Marissa Krizelda D. Santos

**Affiliations:** a Department of Otolaryngology Head and Neck Surgery, Linkou Chang Gung Memorial Hospital, Taoyuan, Taiwan; b Department of Otolaryngology Head and Neck Surgery, St. Luke’s Medical Center, Manila, Philippines; c College of Medicine, Chang Gung University, Taoyuan, Taiwan; d Department of Pathology, Linkou Chang Gung Memorial Hospital, Taoyuan, Taiwan; e Department of Pathology, Chinese General Hospital and Medical Center, Manila, Philippines

**Keywords:** case report, hemilaryngectomy, larynx, mucinous adenocarcinoma

## Abstract

**Introduction and importance:**

This paper emphasizes on an infrequent case of primary laryngeal mucinous adenocarcinoma. Despite partial laryngectomies being a traditional surgical technique, it is not frequently used in the treatment of laryngeal lesions. We would like to highlight the efficacy of open partial laryngectomy as a viable treatment option in cases of nonsquamous cell carcinoma of the larynx. The use of traditional and modern surgical techniques can aid in the preservation of voice and swallowing functions. This case report has been reported in line with the SCARE Criteria [Sohrabi C, Mathew G, Maria N, Kerwan A, Franchi T, Agha RA. The SCARE 2023 guideline: updating consensus Surgical CAse REport (SCARE) guidelines. Int J Surg Lond Engl. 2023;109(5):1136]

**Presentation of the case:**

This paper focuses on a 45-year-old female who consulted at a tertiary private hospital. She had no known medical comorbidities or family history of cancer who complained on persistent globus sensation and frequent throat clearing. Her symptoms subjectively were similar to acid reflux but on flexible endoscopy a laryngeal lesion was seen. The initial impression with the additional information gathered from a neck CT scan was that of a cystic lesion. During surgery, no cystic component was seen; tissue samples were sent for biopsy. The biopsy results were signed out as a case of mucinous adenocarcinoma. Given the age and medical status of the patient, an organ preservation surgery was done. Post operatively the patient was able to maintain all swallowing and phonatory capabilities.

**Clinical discussion:**

There are currently two reported cases of primary laryngeal mucinous adenocarcinoma. Both cases were located in the supraglottic region presenting with dysphonia and dysphagia. Due to the limited number of cases, there is currently no standardized surgical or medical treatment for the management primary laryngeal mucinous adenocarcinomas.

**Conclusion:**

Mucinous adenocarcinoma of the larynx is a rare disease usually found as distant metastatic lesions. Primary laryngeal mucinous adenocarcinoma is even more uncommon. To balance the oncological outcomes and life quality is important. We reported a primary laryngeal mucinous laryngeal adenocarcinoma after combined transoral transcervical partial laryngectomy that with good functional and oncological outcomes.

## Introduction

Primary mucinous adenocarcinomas is a rarely seen in the head and neck region and even more so in the larynx. Among the subsites of the larynx, this particular condition is more commonly identified in the supraglottic region due to the high vascularity and lymphatics. Although this area is a common site for metastasis of mucinous adenocarcinomas, primary mucinous adenocarcinomas of the larynx are considered to be low grade and nonaggressive. Early detection aids in optimizing organ preservation treatments such as partial laryngectomies.

**Highlights**

- To present a case of Laryngeal Mucinous Adenocarcinoma in a young woman.
- To discuss the diagnostic and therapeutic dilemmas involved.
- This case report shares the team’s experience in combining both old and new techniques (hemilaryngectomy and robotic surgery) to preserve the patient’s speech and swallowing functions.
- The case report shares how the patient retains her quality of life after treatment.

## Case report

**Figure 1.**
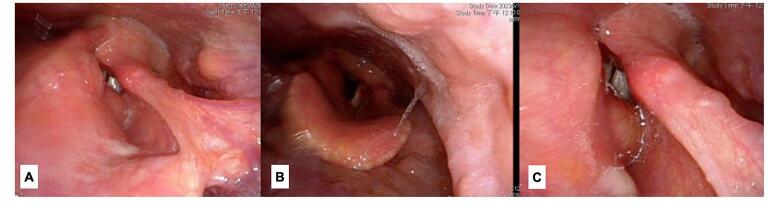
Transnasal flexible endoscopy images: (A) laryngeal view; (B) abduction view; (C) adduction view.

A 45-year-old female patient consulted at the outpatient department of a tertiary private hospital. She presented with a 1-year history of persistent globus sensation with an urge to frequently clear her throat. She did not complain of dysphagia, odynophagia, dyspnea, or weight loss. She has no known medical conditions or pertinent family medical history. A transnasal flexible endoscopy was performed and showed swelling along the right ary-epliglottic fold extending towards the false vocal cords, right arytenoids and right vallecula (Fig. 1). The true vocal cords and cricoarytenoid appeared to be unaffected with smooth vocal fold mucosa and no visible vocal fold lesions. Bilateral vocal folds were movable with symmetric vibration under stroboscopy (Supplementary Digital Content, Video Clip 1 http://links.lww.com/MS9/A727).

**Figure 2.**
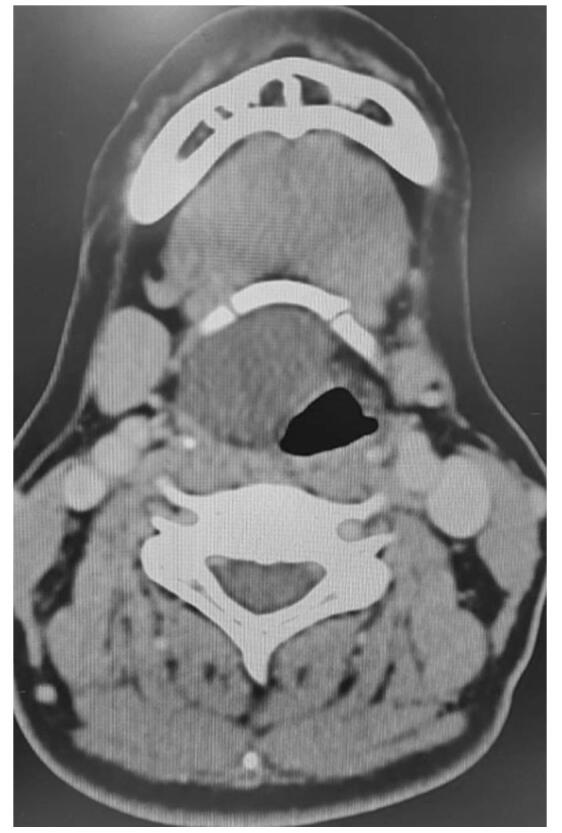
Axial view of neck CT scan: 3.4 × 2.5 × 3.2 cm well-defined hypodense poorly-enhanced nodule at right epiglottis effaced vallecula.

Under the impression of submucosal laryngeal tumor, a neck CT scan with contrast was subsequently requested. The requested CT scan revealed a 3.4 × 2.5 × 3.2 cm well-defined hypodense non-enhanced mass at right epiglottis with prelaryngeal space involvement (Fig. 2).

**Figure 3.**
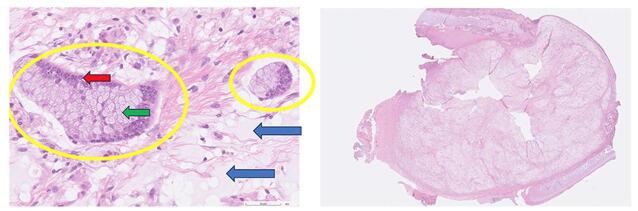
Photomicrograph of specimen submitted: Neoplastic columnar epithelium (yellow circle) are floating in extracellular mucin pools (blue arrows), columnar cells have basally-located nuclei (red arrow) with intracellular mucin (green arrow).

**Figure 4.**
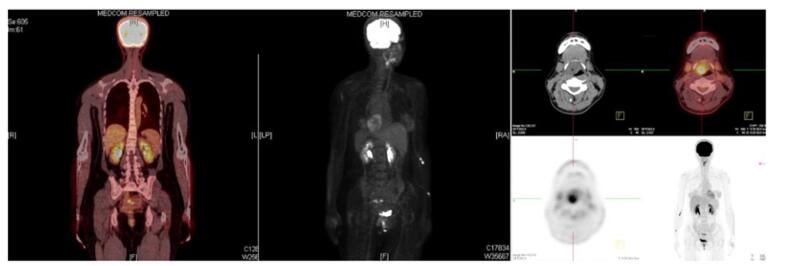
Positron emission test scan.

The patient was then scheduled for a transoral excision of the suspected cystic tumor. However, intraoperatively, the lesion contained hard, whitish lime-like and nonvascular material. There was no fluid content. Samples were sent repeatedly for frozen section analysis, however, no viable cells could be identified. The procedure was then discontinued due to insufficient data from the frozen section. Complete resection was postponed until the final histopathology report was released. The final histologic exam showed obliteration of normal laryngeal tissue, replaced by extracellular mucin pools. Malignant columnar epithelium floating in large pools of extracellular mucin was appreciated yielding a diagnosis of mucinous adenocarcinoma (Fig. 3). Concern of a metastatic lesion was raised hence immunohistochemical staining was requested which included CDX2, and CA 19-9 immunohistochemical staining were requested. The stains yielded the following results: CDX2 negative and CA 19-9 positive; further testing was required to confirm the diagnosis; radiologic and clinical examinations were negative for metastasis from an alternative primary site, hence the diagnosis of a Primary Laryngeal Mucinous Adenocarcinoma, Stage II (Fig. 4).

**Figure 5.**
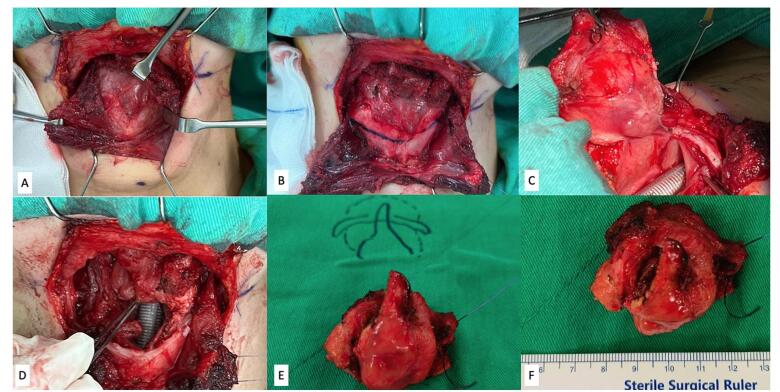
(A) Subplatysmal flap was created and raised; strap muscles were retracted laterally. (B) The midportion of the thyroid cartilage was identified. (C) Prelaryngeal space was removed. (D) Anterior view of surgical defect. (E) Anterior view of gross specimen including the prelaryngeal space, a portion of the epiglottic and a portion of the epiglottis. (F) Superior view of gross specimen including the prelaryngeal space, a portion of the epiglottic and a portion of the epiglottis.

**Figure 6.**
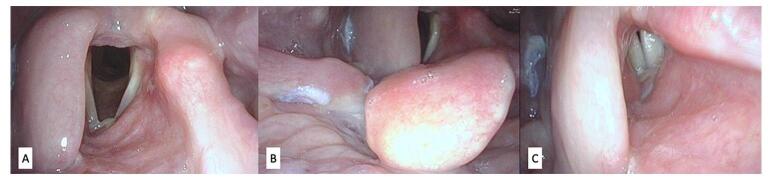
Post-operative transnasal flexible endoscopy (A), abduction view (B), and adduction view (C).

As the patient’s laryngeal function was not affected, she was scheduled for an organ preservation surgery. The patient underwent transoral and transcervical partial supraglottic laryngectomy with tracheostomy (Fig. 5). The later was done for better access and airway support intraoperatively. The procedure was performed by a senior otolaryngologist who specializes in laryngology and head and neck surgery. Initially, via the transoral approach, the mucosal incision was performed through right preepiglottic space to aryepiglottic fold. The surgical field was then shifted to the external neck. A transverse skin incision at the level of thyrohyoid junction was created. The right sternohyoid muscle was divided and retracted to expose the thyroid cartilage. After the lateral part of sternohyoid muscle was freed from the hyoid bone and ligation of the superior laryngeal vessels was completed, the upper third of the right thyroid cartilage lamina and right half of hyoid bone was thoroughly cut. The pharyngeal mucosa was then exposed after mobilizing of the bony laryngeal structure and connected with the intraoral incision line. The prelaryngeal space was then opened and the tumor was removed with adequate safety mucosal margins (Fig. 6). At this point, a neck dissection was not performed due to the absence of enhancing patterns on PET scan outside of the right laryngeal area. The pharyngeal mucosa was then primarily approximated layer by layer.

Four weeks after the operation, the patient was successfully decannulated and was weaned off nasogastric tube feeding. She is able to perform her activities of daily living with no difficulty. The patient adhered to the postoperative instructions which included NGT feeding and gradual transition to oral feeding with strict aspiration precautions. She was able to get support from the medical team, family and friends. The patient is still being followed up quarterly with repeat transnasal flexible laryngoscopies and annual CT scans. After 12 months follow-up there is no recurrence of the malignancy with an intact laryngopharyngeal function (Supplementary Digital Content, Video Clip 2 http://links.lww.com/MS9/A728). There were no untoward events during the management of this case. The patient’s and medical team’s post-operative goals of organ preservation have been fully met.

## Discussion

Adenocarcinomas arising from the mucous glands is a rare disease comprising of 0.35% to 0.5% of all laryngeal malignancies. In nonspecific type of adenocarcinomas, it is more commonly seen in males in the 6th to 7th decade of life^[1,2].^ According to the 2019 World Health Organization Classification (Tumors of the Digestive System 5th edition), mucinous adenocarcinomas are defined as tumors with abundant amounts of extracellular mucin secretion involving more than 50% of its volume with a high risk of lymphatic and distant organ metastasis^[3,4]^. In a population-based study by Xie *et al*, the 5-year survival rate of patient diagnosed with localized mucinous adenocarcinoma is 92.9%^[5]^.

The histogenesis of laryngeal adenocarcinomas up to this date is not well understood but is believed to be due to the overexpression of MUC2. MUC2 is a principal marker in various mechanisms and secretory cell lineage; alterations in its expression are associated with immunomodulation, differences in tumor immunity, and regulation of microbiota. MUC2 has been seen to be related to the decrease in the activation of adaptive and innate immune responses.^[6,7]^ The most common location for mucinous adenocarcinoma in the larynx, also as in the presenting case, is the supraglottic region. It is due to the abundant blood supply compared to the glottic and subglottic regions; the supraglottic region comprises of 35% to 40% of all laryngeal malignancies^[8]^.

Mucinous adenocarcinomas most commonly occur in the gastrointestinal tract and may also be seen in other areas such as the breast, skin, thyroid gland, prostate, and sinonasal tract^[1,2,5,7]^. Primary laryngeal mucinous adenocarcinoma is extremely rare with only two cases previously reported. It is of utmost importance to differentiate primary from metastatic laryngeal mucinous adenocarcinomas. In our case, a positron emission tomography scan and immunohistochemical stainings were performed to exclude the possibility of a distant metastatic lesion from other organs. For this case, a CDX2 was performed to rule out a gastrointestinal tract tumor while CA 19-9 was performed to rule out pancreatic tumor.

To maximize the balance between oncologic control and quality of life is the ideal goal of treatment for laryngeal malignancies. To date, there is no definite treatment for mucinous adenocarcinoma of the larynx. Unlike laryngeal squamous cell carcinomas, radiation therapy was not proposed to be effective for laryngeal mucinous adenocarcinoma^[9]^. In addition, it is believed that cells exhibiting intrinsic radio-resistance persist following radiation therapy and could lead to increased clinical risks due to their revived proliferation and successful colonization at local or distant sites^[9,10]^. Reported cases of laryngeal mucinous adenocarcinoma typically present with symptoms such as dyspnea or dysphonia. These cases commonly involve lesions in the supraglottic area. In one instance as described by Tsang *et al*, a patient presented with a mass along the right side of the epiglottis and a fixed right vocal cord, while another described by Ebru *et al*, exhibited a mass on the left pyriform sinus, epiglottis, right thyroid and left cervical lymphadenopathies. The two previously reported cases of primary laryngeal mucinous adenocarcinoma had accepted total laryngectomy, with and without adjuvant radiation therapy^[1,2]^.

Vertical partial laryngectomy, although is considered as an old surgical technique, is indicated for early to moderate stage laryngeal malignancies.^[11]^ In the era of organ preservation treatment, the role of partial laryngectomy has been changed by the popularization of chemoradiation and endoscopic surgery.^[10,11]^ Considering our patient’s age, functional capabilities and overall health, we opted for a hybrid transoral and transcervical vertical partial laryngectomy. The patient has returned to her premorbid function.

## Conclusion

Primary laryngeal mucinous adenocarcinoma is extremely rare. Functional preservation laryngeal surgery is appropriate for managing laryngeal mucinous adenocarcinoma. This procedure demonstrates efficacy in achieving oncological control while minimizing the impact on vocal function, highlighting its potential as a valuable treatment option in the multidisciplinary management of this specific type of laryngeal cancer. However, individual patient characteristics and tumor staging should be thoroughly evaluated to determine the suitability of this technique, emphasizing the importance of a personalized approach in optimizing outcomes for patients with mucinous adenocarcinoma of the larynx.

## Data Availability

Not applicable.
